# Supplementary material for: Infant and young child feeding practices and its associated factors among mothers of under two years children in a western hilly region of Nepal
Source: PLoS One. 2021 Dec 16;16(12):e0261301. doi: 10.1371/journal.pone.0261301 (PMC8675745; doi:10.1371/journal.pone.0261301)
Supplement: S1 File — (DOCX) [file pone.0261301.s001.docx]

Namaste!

My name is **Nabin Adhikari**. I am a student of Master of Public Health Nutrition (MPHN) studying at Central Department of Public Health, IoM, Kathmandu. As a part of my study, I am conducting a thesis on a topic **“Infant and Young Child Feeding Practices and Its Association with Maternal Factors among Mothers of Under Two Years Children in Syangja District”** for the partial fulfillment of my master’s degree. This study aims to access infant and young child feeding practices and the associated factors of mothers for their children. You have to answer a few questions about your socio-demographic and economic conditions and information regarding feeding practices. Therefore, I would like to request your participation in the study.

Participation in this study is voluntary. You can choose not to answer any individual question or all of the questions. The information gathered from this study will be used for study purpose only. I assure you that all the information provided by you will be kept strictly confidential and will be used only for research purpose. Your name or other facts that might identify you will not appear when we report the findings of this study and I will highly acknowledge your contribution to the study in general. I will also assure you that there are no risks associated with taking part in this study.

If you have any queries regarding the study, feel free to ask and clarify your doubts.

If you agree or do not to participate in this study, I hereby seek your consent.

Agree to participate

Don not agree to participate

|  |  |
| --- | --- |
| **Right** | **Left** |

Signature……………………

Date…………………………
