# Supplementary material for: Infant and young child feeding practices and its associated factors among mothers of under two years children in a western hilly region of Nepal
Source: PLoS One. 2021 Dec 16;16(12):e0261301. doi: 10.1371/journal.pone.0261301 (PMC8675745; doi:10.1371/journal.pone.0261301)
Supplement: S2 File — (DOCX) [file pone.0261301.s002.docx]

**Conceptual framework**

Mother’s Autonomy

- household decision making
- health care decision making
- financial autonomy
- social/mobility autonomy

Socio - demographic and socio-economic variable

Infant and young child feeding practice

- Early initiation of Breastfeeding
- Exclusive breastfeeding
- Introduction of complementary feeding
- Minimum acceptable diet

Maternal health services

- ANC visit
- Place of Delivery
- PNC visit

Mother’s knowledge

- Maternal knowledge about recommended practice
