# Supplementary material for: Infant and young child feeding practices and its associated factors among mothers of under two years children in a western hilly region of Nepal
Source: PLoS One. 2021 Dec 16;16(12):e0261301. doi: 10.1371/journal.pone.0261301 (PMC8675745; doi:10.1371/journal.pone.0261301)
Supplement: S3 File — (DOCX) [file pone.0261301.s003.docx]

**Table 1. Association of independent variables with timely initiation of breastfeeding**

|  | **Timely initiation of breastfeeding**  **n (%)** | |  |  |
| --- | --- | --- | --- | --- |
| **Variables** | **Yes** | **No** | **ꭓ2** | **p- value** |
| **Sex of children** |  |  |  |  |
| Male | 120(67.8) | 57 (32.2) | 0.330 | 0.566 |
| Female | 118 (70.7) | 49 (29.3) |  |  |
| **Age of mothers (year)** |  |  |  |  |
| 15-24 years | 92(71.3) | 37 (28.7) | 0.539 | 0.764 |
| 20-34 years | 139(68.1) | 65(31.9) |  |  |
| ≥ 35 years | 7 (63.6) | 4 (36.4) |  |  |
| **Mother occupation** |  |  |  |  |
| Agriculture | 135(72.2) | 52(27.8) | 1.737 | 0.187 |
| Non agriculture | 103(65.6) | 54(34.4) |  |  |
| **Ethnicity** |  |  |  |  |
| Brahmin | 109(69.4) | 48 (30.6) | 0.008 | 0.929 |
| Non Brahmin | 129 (69) | 58 (31) |  |  |
| **Family type** |  |  |  |  |
| Single | 118 (76.1) | 37 (23.9) | 6.379 | 0.012* |
| Joint | 120(63.5) | 69 (36.5) |  |  |
| **Education** |  |  |  |  |
| Illiterate | 10 (66.7) | 5 (33.3) | 0.600 | 0.741 |
| Informal or primary | 228 (69.3) | 101 (30.7) |  |  |
| Secondary or above | 180(70.3) | 76(29.7) |  |  |
| **Religion** |  |  |  |  |
| Non Hindu | 19 (76) | 6 (24) | 0.587 | 0.444 |
| Hindu | 219 (68.7) | 100 (31.3) |  |  |
| **Child number** |  |  |  |  |
| 1 | 85(66.9) | 42(33.1) | 0.372 | 0.542 |
| 2 | 107(69.9) | 46(30.1) |  |  |
| 3 | 41(73.2) | 15(26.8) |  |  |
| 4 or more | 5(62.5) | 3(37.5) |  |  |
| **Crop production and food security** |  |  |  |  |
| No crop produces or food not enough for 12 months | 74(72.5) | 28(27.5) | 0.769 | 0.380 |
| Produce crop and food enough for 12 months | 164(67.8) | 78(32.2) |  |  |
| **Wealth quintile** |  |  |  |  |
| Lowest | 44 (62) | 27 (38) | 3.917 | 0.417 |
| Second | 54 (77.1) | 16 (22.9) |  |  |
| Middle | 47 (69.1) | 21 (30.9) |  |  |
| Fourth | 46 (67.6) | 22 (32.4) |  |  |
| Highest | 47 (70.1) | 20 (29.9) |  |  |
| **ANC visit** |  |  |  |  |
| None | 18(64.3) | 10 (35.7) | 0.674 | 0.714 |
| 1-3 times | 104(71.2) | 42(28.8) |  |  |
| 4 or more | 116(68.2) | 54(31.8) |  |  |
| **Types of delivery** |  |  |  |  |
| Normal vaginal | 230 (74.2) | 80 (25.8) | 36.891 | <0.001* |
| Cesarean Section | 8 (23.5) | 26 (76.5) |  |  |
| **Delivery place** |  |  |  |  |
| Home | 39(66.1) | 20(33.9) | 0.318 | 0.573 |
| Institutional | 199(69.8) | 86(30.2) |  |  |
| **knowledge on initiation of breastfeeding time** |  |  |  |  |
| Incorrect | 7 (26.9) | 19(73.1) | 23.565 | <0.0001* |
| Correct | 87(27.4) | 231(72.6) |  |  |
| **Autonomy** |  |  |  |  |
| Lowest | 70 (64.8) | 38 (35.2) | 5.462 | 0.065 |
| Middle | 79 (65.3) | 42 (34.7) |  |  |
| Highest | 89 (77.4) | 26 (22.6) |  |  |

**Table 2. Association of independent variables with exclusive breastfeeding practice**

|  | **Exclusive breastfeeding**  **n (%)** | |  |  |
| --- | --- | --- | --- | --- |
| **Variables** | **Yes** | **No** | **ꭓ2** | **p- value** |
| **Sex of child** |  |  |  |  |
| Male | 25(47.2) | 28(52.8) | 0.007 | 0.933 |
| Female | 24(48) | 26(52) |  |  |
| **Age of mother (years)** |  |  |  |  |
| 15-24 years | 17(41.5) | 24(58.5) | 1.021 | 0.600 |
| 25-34 years | 31(51.7) | 29 (48.3) |  |  |
| ≥ 35 | 1 (50) | 1 (50) |  |  |
| **Ethnicity** |  |  |  |  |
| Brahmin/Chettri | 27 (50) | 27 (50) | 0.268 | 0.605 |
| Non Brahmin/chettri | 22 (44.9) | 27 (55.1) |  |  |
| **Family type** |  |  |  |  |
| Single | 22 (53.7) | 19 (46.3) | 1.011 | 0.315 |
| Joint | 27 (43.5) | 35 (56.5) |  |  |
| **Education** |  |  |  |  |
| Informal and primary | 9 (40.9) | 13 (59.1) | 0.498 | 0.480 |
| Secondary and above | 40 (49.4) | 41 (50.6) |  |  |
| **Religion** |  |  |  |  |
| Non-Hindu | 6(75) | 2(25) | 2.616 | 0.146 |
| Hindu | 43(45.3) | 52(54.7) |  |  |
| **Occupation** |  |  |  |  |
| Agriculture | 29 (53.7) | 25 (46.3) | 1.711 | 0.191 |
| Non agriculture | 20 (40.8) | 29 (59.2) |  |  |
| **Child number** |  |  |  |  |
| 1 | 15(39.5) | 23(60.5) | 2.223 | 0.329 |
| 2 | 28(54.9) | 23(45.1) |  |  |
| 3 | 6(42.9) | 8(57.1) |  |  |
| 4 or more | 0 | 0 |  |  |
| **Crop production and food security** |  |  |  |  |
| No crop produces or food not enough for 12 months | 18(40) | 27(60) | 1.837 | 0.175 |
| Produce crop and food enough for 12 months | 31(53.4) | 27(46.6) |  |  |
| **Wealth quintile** |  |  |  |  |
| Lowest | 4 (30.8) | 9 (69.2) | 3.492 | 0.479 |
| Second | 13 (59.1) | 9 (40.9) |  |  |
| Middle | 10 (45.5) | 12 (54.5) |  |  |
| Fourth | 13 (54.2) | 11 (45.8) |  |  |
| Highest | 9 (40.9) | 13 (59.1) |  |  |
| **ANC visit** |  |  |  |  |
| None | 2(40) | 3(60) |  |  |
| 1-3 times | 25(55.6) | 20(44.4) | 2.051 | 0.359 |
| 4 or more | 22(41.5) | 31(58.5) |  |  |
| **Delivery place** |  |  |  |  |
| Home delivery | 11(68.8) | 5(31.2) | 3.406 | 0.065 |
| Instutional delivery | 38(43.7) | 49(56.3) |  |  |
| **PNC visit** |  |  |  |  |
| None | 17(63) | 10(37) |  |  |
| One time | 15 (37.5) | 25 (62.5) | 3.475 | 0.062 |
| Two or more time | 17 (47.2) | 19 (52.8) |  |  |
| **Types of delivery** |  |  |  |  |
| Normal | 47 (51.1) | 45 (48.9) | 4.265 | 0.039* |
| Cesarean section | 2 (18.2) | 9 (81.8) |  |  |
| **Growth monitoring** |  |  |  |  |
| Yes | 38 (46.9) | 43 (53.9) | 0.066 | 0.797 |
| No | 11 (50) | 11 (50) |  |  |
| **Counseling on nutrition** |  |  |  |  |
| Yes | 28 (48.3) | 30 (51.7) | 0.026 | 0.871 |
| No | 21 (46.7) | 24 (53.3) |  |  |
| **Knowledge on exclusive breastfeeding** |  |  |  |  |
| Incorrect | 4 (30.8) | 9 (69.2) | 1.684 | 0.194 |
| Correct | 45 (50) | 45 (50) |  |  |
| **Maternal Autonomy** |  |  |  |  |
| Lowest | 9 (27.3) | 24 (72.2) | 9.229 | 0.010* |
| Middle | 16 (50) | 16 (50) |  |  |
| Highest | 24 (63.2) | 14 (36.8) |  |  |

**Table 3. Association of independent variable with timely initiation of complementary feeding**

|  | **Timely initiation of complementary feeding n (%)** | |  |  |
| --- | --- | --- | --- | --- |
| **Variables** | **Yes** | **No** | **ꭓ2** | **p- value** |
| **Sex** |  |  |  |  |
| Male | 96 (72.7) | 36 (27.3) | 58.998 | 0.001* |
| Female | 31 (26.4) | 94 (73.6) |  |  |
| **Age of mother (years)** |  |  |  |  |
| 15-24 years | 51(52.6) | 46 (47.4) | 3.061 | 0.216 |
| 25-34 years | 74 (49) | 77 (51) |  |  |
| ≥ 35 years | 2 (22.2) | 7 (77.8) |  |  |
| **Ethnicity** |  |  |  |  |
| Non-Brahmin/chhettri | 60 (52.6) | 54 (47.4) | 0.847 | 0.357 |
| Brahmin/chhettri | 67 (46.9) | 76 (53.1) |  |  |
| **Occupation** |  |  |  |  |
| Non-Agriculture | 63(53.4) | 55(46.6) | 1.378 | 0.240 |
| Agriculture | 64(46) | 75(54) |  |  |
| **Religion** |  |  |  |  |
| Non-Hindu | 9 (50) | 9 (50) | 0.003 | 0.959 |
| Hindu | 118 (49.4) | 121 (50.6) |  |  |
| **Education** |  |  |  |  |
| Illiterate | 6(37.5) | 10(62.5) | 1.395 | 0.498 |
| Informal or primary | 25(46.3) | 29(53.7) |  |  |
| Secondary or above | 96(51.3) | 91(48.7) |  |  |
| **Types of family** |  |  |  |  |
| Single | 57(48.3) | 61(51.7) | 0.108 | 0.743 |
| Joint | 70(50.4) | 69(49.6) |  |  |
| **Child Number** |  |  |  |  |
| 1 | 52(54.7) | 43(45.3) | 6.242 | 0.012* |
| 2 | 60(55) | 49(45) |  |  |
| 3 | 11(24.4) | 34(75.6) |  |  |
| 4 or more | 4(50) | 4(50) |  |  |
| **Crop production and food security** |  |  |  |  |
| No crop produces or food not enough for 12 months | 32(50.8) | 31(49.2) | 0.063 | 0.801 |
| Produce crop and food enough for 12 months | 95(49) | 99(51) |  |  |
| **Wealth quintile** |  |  |  |  |
| Lowest | 24 (40.7) | 35 (59.3) | 2.820 | 0.588 |
| Second | 27 (54) | 23 (46) |  |  |
| Middle | 27 (62) | 23 (46) |  |  |
| Fourth | 23 (47.9) | 25 (52.1) |  |  |
| Highest | 26 (52) | 24 (48) |  |  |
| **ANC visit** |  |  |  |  |
| None | 7 (29.2) | 17 (70.8) | 4.427 | 0.109 |
| 1-3 times | 54(50.5) | 53(49.5) |  |  |
| 4 or more times | 66(52.4) | 60(47.6) |  |  |
| **Delivery place** |  |  |  |  |
| Home | 15 (34.1) | 29 (65.9) | 4.988 | 0.026* |
| Institution | 112 (52.6) | 101 (47.4) |  |  |
| **Delivery type** |  |  |  |  |
| Normal | 117 (50.9) | 113 (49.1) | 1.850 | 0.174 |
| Cesarean Section | 10 (37) | 17 (63) |  |  |
| **PNC visit** |  |  |  |  |
| None | 22 (41.5) | 31 (58.5) | 1.670 | 0.434 |
| 1 time | 54(51.4) | 51(48.6) |  |  |
| 2 or more | 51(51.5) | 48(48.5) |  |  |
| **Growth monitoring** |  |  |  |  |
| Yes | 94 (54.7) | 78 (45.3) | 5.701 | 0.017* |
| No | 33 (38.8) | 52 (61.2) |  |  |
| **Counseling on nutrition** |  |  |  |  |
| Yes | 61(59.2) | 42(40.8) | 6.614 | 0.010* |
| No | 66(42.9) | 88 (57.1) |  |  |
| **Knowledge on initiation of semisolid food** |  |  |  |  |
| Incorrect | 12(33.3) | 24(66.7) | 4.332 | 0.037 |
| Correct | 115(52) | 106(48) |  |  |
| **Autonomy** |  |  |  |  |
| Lowest | 35 (42.7) | 47 (57.3) | 2.820 | 0.244 |
| Middle | 51 (55.4) | 41 (44.6) |  |  |
| Highest | 41 (49.4) | 42 (50.6) |  |  |

**Table 4. Association of independent variables with minimum acceptable diet (MAD)**

|  | **Meet MAD**  **n (%)** | |  |  |
| --- | --- | --- | --- | --- |
| **Variables** | **Yes** | **No** | **ꭓ2** | **p- value** |
| **Sex of children** |  |  |  |  |
| Male | 60 (45.5) | 72 (54.5) | 1.704 | 0.192 |
| Female | 67 (53.6) | 58 (46.4) |  |  |
| **Age of mother (years)** |  |  |  |  |
| 15-24 | 45(46.4) | 52(53.6) | 0.918 | 0.632 |
| 25-34 | 77(51) | 74(49) |  |  |
| ≥ 35 | 5(55.6) | 4(44.4) |  |  |
| **Ethnicity** |  |  |  |  |
| Brahmin/chhettri | 71(49.7) | 72(50.3) | 0.007 | 0.933 |
| Non Brahmin/chhettri | 56(49.1) | 58(50.9) |  |  |
| **Family type** |  |  |  |  |
| Single | 57 (48.3) | 61 (51.7) | 0.108 | 0.743 |
| Joint | 70 (50.4) | 69 (49.6) |  |  |
| **Religion** |  |  |  |  |
| Hindu | 114 (47.7) | 125 (52.3) | 4.027 | 0.054 |
| Non-Hindu | 13 (66.7) | 5 (33.3) |  |  |
| **Education** |  |  |  |  |
| Illiterate | 6(37.5) | 10(62.5) | 5.799 | 0.055 |
| Informal or primary | 20 (37) | 34(63) |  |  |
| Secondary or above | 101 (54) | 86(46) |  |  |
| **Occupation** |  |  |  |  |
| Agriculture | 61(43.9) | 78(56.1) | 3.706 | 0.054 |
| Non agriculture | 66 (55.9) | 52 (44.1) |  |  |
| Child number |  |  |  |  |
| 1 | 51(53.7) | 44(46.3) | 3.179 | 0.365 |
| 2 | 55(50.5) | 54(49.5) |  |  |
| 3 | 17(37.8) | 28(62.6) |  |  |
| 4 or more | 4(50) | 4(50) |  |  |
| **Crop production and food security** |  |  |  |  |
| No crop produces or food not enough for 12 months | 15(23.8) | 48(76.2) | 21.893 | <0.001* |
| Produce crop and food enough for 12 months | 112(57.7) | 82(42.3) |  |  |
| **Wealth quintile** |  |  |  |  |
| Lowest | 25 (42.4) | 34 (57.6) | 8.192 | 0.085 |
| Second | 23 (46) | 27 (54) |  |  |
| Middle | 26 (52) | 24 (48) |  |  |
| Fourth | 20 (41.7) | 28 (58.3) |  |  |
| Highest | 33 (66) | 17 (34) |  |  |
| **ANC visit** |  |  |  |  |
| None | 8 (33.3) | 16 (66.7) | 2.927 | 0.231 |
| 1-3 times | 53(49.5) | 54(50.5) |  |  |
| 4 or more times | 66(52.4) | 60(47.6) |  |  |
| **Delivery place** |  |  |  |  |
| Home | 15(34.1) | 29(65.9) | 4.988 | 0.026* |
| Institution | 112(52.6) | 101(47.4) |  |  |
| **PNC visit** |  |  |  |  |
| None | 18 (34) | 35 (66.0) | 6.475 | 0.039* |
| 1 time | 55(52.4) | 50(47.6) |  |  |
| 2 or more times | 54(54.5) | 45(45.5) |  |  |
| **Delivery type** |  |  |  |  |
| Normal | 110(47.8) | 120(52.5) | 2.215 | 0.137 |
| Cesarean Section | 17(63) | 10(37) |  |  |
| **Growth monitoring** |  |  |  |  |
| Yes | 91 (52.9) | 81 (47.1) | 2.535 | 0.111 |
| No | 36 (42.4) | 49 (57.6) |  |  |
| **Counseling on nutrition** |  |  |  |  |
| Yes | 62 (60.2) | 41 (45.5) | 7.988 | 0.005* |
| No | 65 (42.2) | 89 (57.8) |  |  |
| **Knowledge on MAD** |  |  |  |  |
| Incorrect | 106(46.9) | 120(53.1) | 4.763 | 0.030* |
| Correct | 21(67.7) | 10(32.3) |  |  |
| **Autonomy** |  |  |  |  |
| Lowest | 22 (26.3) | 60 (73.2) | 25.297 | <0.001* |
| Middle | 58 (63) | 34 (37) |  |  |
| Highest | 47 (56.6) | 36 (43.4) |  |  |
