# Supplementary material for: Infant and young child feeding practices and its associated factors among mothers of under two years children in a western hilly region of Nepal
Source: PLoS One. 2021 Dec 16;16(12):e0261301. doi: 10.1371/journal.pone.0261301 (PMC8675745; doi:10.1371/journal.pone.0261301)
Supplement: S1 Questionnaire — (DOCX) [file pone.0261301.s004.docx]

| Information panel  (this information is entered after identifying eligible children from the household) | | | | | | | | | |
| --- | --- | --- | --- | --- | --- | --- | --- | --- | --- |
| Date: __ __/__ __/__ __ __ __  Day Month Year | | | | | | | | | |
| Form No.: | | | | | | | | | |
| Name of municipality: | | | | | | | | | |
| Ward No.: | | | | | | | | | |
|  | What is your young child name?  Is (NAME) male or female? | | …………………………..  Male  Female | | | | | 1  2 |  |
|  | When did Name born? | | Birth date:_ _ /_ _/_ _ _ _  dd/mm/year  Age in completed months………………  Birth date from birth certificate, vaccination card, remember by mother | | | | | |  |
| **Section 1: demographic and socioeconomic information** | | | | | | | | | |
| **S.N.** | **Questions** | | **Options** | | | | | **Coding** | **Remarks** |
| 1 | Age of mother in completed years | |  | Years | | | |  |  |
| 2 | Ethnicity of mother | | Brhamin  Chettri  Janajati  Dalit | | | | | 1  2  3  4 |  |
| 3 | Type of family | | Nuclear  Joint  Extended | | | | | 1  2  3 |  |
| 4 | What is your religion? | | Hindu  Buddhist  Muslim  Christian  Others (specify)………… | | | | | 1  2  3  4  96 |  |
| 5 | What is your Education level | | Illiterate  Literate only  Specify grade if had formal education | | | | | 1  2  …. |  |
| 6 | What is your occupation? | | Agriculture  Labor  Service  Business  Housewife  Others (specify)…………. | | | | | 1  2  3  4  5  96 |  |
| 7 | Do you work outside from house? | | Yes  No | | | | | 1  0 |  |
| 8 | How many hours do you spent in work? | | -------------- hrs | | | | |  |  |
| 9 | Does anyone help in your work so you could look after (NAME) | | Yes  No | | | | | 1  0 | In no go q 11 |
| 10 | In what kind of work they help? | | Cooking food | | | | | 1 |  |
|  |  |  | Clean house | | | | | 2 |  |
|  |  |  | Help in kitchen garden | | | | | 3 |  |
|  |  |  | Marketing | | | | | 4 |  |
|  |  |  | Look after cattle | | | | | 5 |  |
|  |  |  | Others (specify) | | | | | 96 |  |
| 11 | How much do you earn in a month? | | Rs………….. | | | | |  |  |
| 12 | How much does your family earn in a month? | | Rs…………… | | | | |  |  |
| 13 | How many children do you have? | | ……… | | | | |  |  |
| 14 | Does your household have | |  | | | | Yes | No |  |
|  |  |  | Electricity | | | | 1 | 0 |  |
|  |  |  | A radio | | | | 1 | 0 |  |
|  |  |  | Television | | | | 1 | 0 |  |
|  |  |  | Telephone | | | | 1 | 0 |  |
|  |  |  | Computer | | | | 1 | 0 |  |
|  |  |  | Refrigerator | | | | 1 | 0 |  |
|  |  |  | Table | | | | 1 | 0 |  |
|  |  |  | Chair | | | | 1 | 0 |  |
|  |  |  | Bed | | | | 1 | 0 |  |
|  |  |  | Sofa | | | | 1 | 0 |  |
|  |  |  | Cupboard | | | | 1 | 0 |  |
|  |  |  | Clock | | | | 1 | 0 |  |
|  |  |  | Fan | | | | 1 | 0 |  |
|  |  |  | An invertor | | | | 1 | 0 |  |
|  |  |  | Dhiki/jhato | | | | 1 | 0 |  |
| 15 | Do you have separate room, which is used as a kitchen? | |  | | | | Yes | No |  |
|  |  |  |  | | | | 1 | 0 |  |
| 16 | Does your household have own any livestock, herds, other farm animal or poultry | |  | | | | 1 | 0 |  |
| 17 | If yes which of following animal does have | | Milk cow | | | | 1 | 0 |  |
|  |  |  | Buffalo | | | | 1 | 0 |  |
|  |  |  | Chicken or other poultry | | | | 1 | 0 |  |
|  |  |  | Cheep | | | | 1 | 0 |  |
|  |  |  | Goat | | | | 1 | 0 |  |
|  |  |  | Horse, donkey, mules | | | | 1 | 0 |  |
|  |  |  | Duck | | | | 1 | 0 |  |
|  |  |  | Pigs | | | | 1 | 0 |  |
|  |  |  | Yaks | | | | 1 | 0 |  |
| 18 | What type of fuel does your household mainly used for cooking | | Electricity | | | | 1 | 0 |  |
|  |  |  | LPG | | | | 1 | 0 |  |
|  |  |  | BIOGAS | | | | 1 | 0 |  |
|  |  |  | Kerosene | | | | 1 | 0 |  |
|  |  |  | Wood | | | | 1 | 0 |  |
|  |  |  | Animal dung | | | | 1 | 0 |  |
|  |  |  | Other (specify ) | | | | 1 | 0 |  |
| 19 | Does any member of this household own any agricultural land? | |  | | | | Yes | No |  |
|  |  |  |  | | | | 1 | 0 |  |
| 20 | Does any member of this household own: | | Watch | | | | 1 | 0 |  |
|  |  |  | Mobile phone | | | | 1 | 0 |  |
|  |  |  | Bicycle | | | | 1 | 0 |  |
|  |  |  | Motorcycle/scooter | | | | 1 | 0 |  |
|  |  |  | Car/truck/tractor | | | | 1 | 0 |  |
|  |  |  | Animal drawn cart | | | | 1 | 0 |  |
|  |  |  | Three wheel tempo | | | | 1 | 0 |  |
| 21 | Does any member of this household have a bank account/cooperative or other savings account? | |  | | | | 1 | 0 |  |
| **Information on Maternal health services related information**  Think back to your last pregnancy | | | | | | | | | |
| 22 | Did you see anyone for antenatal care for this pregnancy? | | Yes  No | | | | | 1  0 | If not go to q.no. 24 |
| 23 | How many times did you receive antenatal care during this pregnancy? | | Number of time  Don’t know | | | | | ……  8 |  |
| 24 | Where did you give birth to (NAME)? | | Home  Health facility  Others (specify)……………. | | | | | 1  2  96 |  |
| 25 | What was the mode of delivery | | Normal delivery  CS | | | | | 1  2 |  |
| 26 | Have you ever visited any health facility for PNC | | Yes  No | | | | | 1  0 | If not go to q.no. 28 |
| 27 | How many times did you visited PNC | | ………………. Times | | | | |  |  |
| 28 | Have you been counseled by any health-related professional (including FCHV) about Maternal, Infant and Young Child Nutrition (MIYCN) in the last 6 months? | | Yes  No | | | | | 1  0 | If not go to Q30 |
| 29 | If yes then on what topic they counseled? | | ………………. | | | | |  |  |
| 30 | Have you done growth monitoring and promotion | | Yes  No | | | | | 1  0 | If no q 32 |
| 31 | Have you counseled about nutrition during growth monitoring | | Yes  No | | | | | 1  0 |  |
| **Feeding infants (0–6 months)**  **Initiation of breastfeeding**  I am going to ask you some questions about nutrition of infants from birth to six months old. Please let me know if you need me to clarify any of my questions. Feel free to ask any question you may have. | | | | | | | | | |
| 32 | Has (NAME) ever been breastfed? | | Yes  No  Don’t know | | | | | 1  0  8 | If no 35 |
| 33 | how long after birth did you first put (NAME) to the breast? | | Immediately within the 1 hrs  Within the 1 st day (1 – 23 hours)  More than 24 hours  Don’t know / Don’t remember | | | | | 1  2  3  8 |  |
| 34 | Did you feed the first milk?  (colostrum)? | | Yes  No  Don’t know | | | | | 1  0  8 |  |
| 35 | If (NAME) did not drink breastmilk what did (name) drink? | | Infant formula  Other milk  Water  Nothing  Other specify | | | | | 1  2  3  4  96 |  |
| **Exclusive breastfeeding**  I am going to ask you some questions about nutrition of infants from birth to six month sold. Please let me know if you need me to clarify any of my questions. Feel free to ask any question you may have. | | | | | | | | | |
| 36 | Was (NAME) breastfed yesterday during the day or at night? | | Yes  No  don’t Know | | | | | 1  0  8 | If no 39 |
| 37 | I would like to ask you about some liquids that (NAME) may have had yesterday during the day or at night.  Did (NAME) have any (ITEM FROM LIST)?: | |  | | Yes | No | | DK |  |
|  |  |  | Plain water | | 1 | 0 | | 8 |  |
|  |  |  | Infant formula | |  |  | |  |  |
|  |  |  | Milk like tinned, powders, animal milk | |  |  | |  |  |
|  |  |  | Juice | |  |  | |  |  |
|  |  |  | Clear broth | |  |  | |  |  |
|  |  |  | Yogurt | |  |  | |  |  |
|  |  |  | Thin porridge | |  |  | |  |  |
|  |  |  | Any other liquid | |  |  | |  |  |
| 38 | For how long did (NAME) give breastmilk only? | | ……… month | | | | |  |  |
| **Feeding young children (6–23 months)**  **Complementary feeding**  I am going to ask you some questions about nutrition of infants aged from 6 to 23 months. Please let me know if you need me to clarify any of my questions. Feel free to ask any question you may have. | | | | | | | | | |
| 39 | Was (*name of the baby*) breastfed or did he or she consume breastmilk yesterday during the day or at night? | | Yes  No  don’t Know | | | | | 1  0  8 |  |
| 40 | When you start the weaning of (NAME)? | | Record exact month | | | | | …… |  |
| 43 | Did (NAME) eat any solid, semi-solid, or soft foods yesterday during the day or at night?  IF ‘YES’ PROBE: What kind of solid, semi-solid or soft foods did (NAME) eat? | | Yes  No | | | | | 1  0 |  |
| 44 | How many times did (NAME) eat solid, semi-solid, or soft foods yesterday during the day or at night? | | Number of times  Don’t know | | | | | __ __  8 |  |
| 45 | Now I would like to ask you about foods that you had yesterday during the day or at night. I am interested in whether you had the item I mention even if it was combined with other foods.  Did you drink or eat: | | | | | | | | |
|  | Group | Food list | Yes | | No | | | DK |  |
| 45.1 | Grains, roots  and tubers | bread, rice, noodles or other foods made from grains  White potatoes, white yams, manioc, cassava or any  other foods made from roots |  | |  | | |  |  |
| 45.2 | Legumes and nuts | Any foods made from beans, peas, lentils, nuts or  Seeds |  | |  | | |  |  |
| 45.3 | Dairy product | Infant formula, Milk such as tinned, powdered or fresh animal milk, Yogurt or drinking yogurt, Cheese or other dairy products |  | |  | | |  |  |
| 45.4 | Meat | Meat product |  | |  | | |  |  |
| 45.5 | Egg | Egg |  | |  | | |  |  |
| 45.6 | Vitamin A fruits and vegetable | Pumpkin, carrots, squash or sweet potatoes, Any dark green vegetables, Ripe mangoes (fresh or dried [not green]), ripe papayas (fresh or dried), and **other**  **local vitamin-A-rich fruits** |  | |  | | |  |  |
| 45.7 | Others fruits and vegetable | Any other fruits or vegetables |  | |  | | |  |  |
| **Section 3: Mothers knowledge on recommended infant and young child feeding Practices** | | | | | | | | | |
| 46 | What is the first food a newborn baby should receive? | | Only breastmilk  Other  Don’t know | | | | | 1  0  88 | Known  Unknown |
| 47 | How long after birth do you think a baby should start breastfeeding | | Within 1 hrs  Others  Don’t know | | | | | 1  0  88 | Known  Unknown |
| 48 | How long should a baby receive nothing more than breastmilk? | | From birth to 6 months  Other  Don’t know | | | | | 1  0  88 | Known  Unknown |
| 49 | How long is it recommended that a woman breastfeeds her child? | | Six months or less  6–11 months  12–23 months  24 months and more  Other  Don’t know | | | | | 1  2  3  4  0  88 | Known  Unknown |
| 50 | At what age should babies start eating foods in addition to breastmilk? | | At six months  Other  Don’t know | | | | | 1  0  88 | Known  Unknown |
| 51 | Please look at these two pictures of porridges. Which one do you think should be given to a young child?  (show picture) | | thick porridge  watery porridge  Does not know | | | | | 1  2  88 | Known  Unknown |
| 52 | In your opinion, what would be the variety of food to be given to 6-23 months aged children? | | Cereals  Pulses  Green leafy vegetables and orange colored fruits  Animal source food such as fish ,meat, eggs, milk, and other milk product  Others (specify)….. | | | | | 1  2  3  4  0 | Known  Unknown |
|  | In your opinion, how many times the child should be feed in 24 hours? | | Meal | | | | | Snack | Remarks |
| 53 | 6 to 8 month | | ………………… | | | | | ………… | Known  Unknown |
| 54 | 9 to 23 month | | ……………….. | | | | | …………. | Known  Unknown |
| **Section 4: Information related to mothers involve in agriculture** | | | | | | | | | |
| 56 | Have you produced..[crop].. during the past 12 months? | | Yes  No | | | | | 1  2 |  |
| 57 | Make a list of crop production in the past 12 months; | | …………………  ………………….  ……………….. | | | | |  |  |
| 58 | How many months in the past 12 months did you consume..[FOOD].. that you grew or produced yourself? (In none write 00) | | ……………… month | | | | |  |  |
| 59 | Does this household have home gardening? | | Yes  No | | | | | 1  2 |  |
|  | **Section 5: information on mother autonomy** | | | | | | | | |
|  | Who will make decision on (on the following occasions) | | | | | | |  |  |
| 61 | Household purchase for daily need | | Own Self  Own self and husband jointly  Husband only  Father or mother in law  Others (specify) | | | | | 5  4  3  2  1 |  |
| 62 | Make large household purchase | | Own Self  Own self and husband jointly  Husband only  Father or mother in law  Others (specify) | | | | | 5  4  3  2  1 |  |
| 63 | Which food to cook or each day | | Own Self  Own self and husband jointly  Husband only  Father or mother in law  Others (specify) | | | | | 5  4  3  2  1 |  |
| 64 | Which food feed for child | | Own Self  Own self and husband jointly  Husband only  Father or mother in law  Others (specify) | | | | | 5  4  3  2  1 |  |
| 65 | Ordered household work | | Own Self  Own self and husband jointly  Husband only  Father or mother in law  Others (specify) | | | | | 5  4  3  2  1 |  |
| 66 | How your inherited asset used | | Own Self  Own self and husband jointly  Husband only  Father or mother in law  Others (specify) | | | | | 5  4  3  2  1 |  |
| 67 | To seek health care for you and your children | | Own Self  Own self and husband jointly  Husband only  Father or mother in law  Others (specify) | | | | | 5  4  3  2  1 |  |
| 68 | To visit your family and friends | | Own Self  Own self and husband jointly  Husband only  Father or mother in law  Others (specify) | | | | | 5  4  3  2  1 |  |
| 69 | To take participate on social activities | | Own Self  Own self and husband jointly  Husband only  Father or mother in law  Others (specify) | | | | | 5  4  3  2  1 |  |

## Thank you
