# Supplementary material for: Infant and young child feeding practices and its associated factors among mothers of under two years children in a western hilly region of Nepal
Source: PLoS One. 2021 Dec 16;16(12):e0261301. doi: 10.1371/journal.pone.0261301 (PMC8675745; doi:10.1371/journal.pone.0261301)
Supplement: Attachment — Submitted filename: Response to Reviewers.docx [file pone.0261301.s006.docx]

**Reviewer #1:** Congratulations on your article! It is notable that it required hard working.

Methods are detailed and make readers understand your trains of thought.

Results are clear, although it would be nice to show data about exclusive breastfeeding in children from 6-23 months. It would be interesting to check if the children exclusively breastfed at age 0-5 months had better feeding practices than children not breastfed. Besides, add information about how long these children were breastfeed would clarify feeding habits of the studied population and all social aspects involved. Data shown on Table 3 relate to exclusive breastfeeding of the 103 children from 0-5 months. Data about the other 237 children would increase the interest on your article and would add relevant information to your research.

**Response**- *Thank you for your comments. As per assessment tools for infant and young child feeding practices we can assess the exclusive breastfeeding of 0-5 months children only so this tools is not valid to assess exclusive breastfeeding in children from 6-23 months.*

Discussion is well written and full of valid references. Comparing your data to data from countries with similar social issues enable readers to understand the range of your article.

**Response**- *Thank you for the comment. We have added relevant references.*

**Reviewer #2: Review**

Title page:

Line 5-24: The authorship order is bit confusing. The lead authorship, corresponding author and senior authorship is not clear. It would be better if the lead or senior author become the corresponding author. Moreover, the equal contribution in the middle is not clear to me. Equal contribution is mostly applicable for lead author and senior author.

**Response**- *Thank you for the comment. We have amended the order of authorship.*

Introduction

1. Line 54-55: When the complementary feeding should be started?

**Response**- *Complementary feeding should be started after six months of age. We have mentioned it now on final version of manuscript*.

2. Line 61-66: Now it is 2021. Is there any updated data available? Please use the updated evidence throughout the Introduction section.

**Response**- *Thank you for the comment. We have now updated the data*.

3. Line 61-72: Concise the write-up. The concept is repeating

**Response**- *Thank you for the comment. We have shortened it*.

4. Line 73-82: Why the autonomy was repeated and emphasized so much? But nothing told about the maternal care seeking practice indicators.

**Response**-*Thank you for the comment. We have now included maternal care seeking related evidence from Nepal.*

5. Line 73-85: The problem identification of necessities to explore the association maternal health seeking practice is not well defined. That means the rationale of the objective is not enough convincing.

**Response**- *Thank you for the comment. We have explored the evidence related to maternal health services and infant and young child feeding practices.*

Here the authors need to mention the available determinates found in the available similar literatures of Nepal and similar countries. Authors also need to find out at least one paper that explored the association of the maternal care seeking factors and child IYCF practices. If no specific paper found then the biological plausibility need to establish by the convincing argument. Hence, the conceptual framework needs to develop with the plausible independent factors. MNH practices could be one of the domains of conceptual framework and others factors will be the covariates/confounders.

**Response**- *Thank you for the comment. We have explored the evidence related to maternal health services and infant and young child feeding practices and provided two references (Reference 6 and 7) for the same. Conceptual framework attached as supplementary file S5.*

Materials and methods

1. Line 98-99: Tell how many of districts 77 district are similar to Syangja and what is the total population of those similar district? Otherwise your sample only represents the limited 289,148 population of one district. Policy makers may not have the importance to the limited number of population. If possible then add a map.

**Response**- *Thank you for the comment. Syangja district is one of the 20 hilly districts of Nepal. Our country has been divided into three ecological zone (i.e. mountain, hill, and terai) based on geographical situation and seventy-seven district based on population size, geographical location. About 45 percent of total population of Nepal live in the hilly districts and we have mentioned this in the methods.*

2. Line 99: 1.164 km2 must be corrected.

**Response**- *Thank you. We have corrected it.*

3. Line 104-106: From how many rural and how many urban municipalities? Is rural administrative unit also called municipality in Nepal? What is the range of number of wards in municipalities?

**Response**- *Thanks for the comment. Altogether there are five urban municipalities and six rural municipalities in Syangja district. Rural administrative unit also called as rural municipality in Nepal. A ward is the smallest administrative unit in Nepal and number of wards in a municipality ranges from minimum of five to maximum of 33. We have mentioned this is the methods.*

4. Line 107-108: Not clear. What is proportionate to population size? Did authors make a list by themselves and randomly select the participants or something else? Please write what actually done there? Sometime the extensive sampling process may not possible to follow for the time and cost constraint. This could be a possible limitation of study.

**Response**- *Thank you for the comment. Proportionate to population size is method of sampling in which required number of samples was identified based on proportion of sample where the probability of selecting a unit is proportional to its size. Authors had access to list of under two years children from health post of the ward and records from female community health volunteers. Author used this list as a sample frame. Regarding municipalities and ward, authors had access to list of municipalities and wards from Provincial Administrative Office.*

5. Line 109-110: Add reference of minimum acceptable diet as 36% and total under two years children in Syangja district as 10524.

**Response**- *Thank you for the comment. Reference has been added*.

6. Line132-155: Please reduce the text. Only MAD was analyzed as main outcome variable. It is better to reduce text throughout outcome variable definitions.
**Response**- *Thank you for the comment. We have done it.*

7. Line 157-160: Need to add citation for all the independent variables. All the factors are the covariates/confounders between maternal health service factors and IYCF. It is better to move this part in Introduction and aligned with the conceptual framework formulation.

**Response**- *Thank you for the comment. We have done this.*

8. Line 174-176: How many researchers collected data? It is very difficult to conduct 360 interviews in 21 days by 1 person. What is the average time of interview?

**Response**- *Thank you for your comment. Data collection team included five trained enumerators and author himself. Average time to taken interview was 45 min. We have mentioned this in the tools section.*

9. Line 173-189: Move the data collection tools section before the variable section. If possible then aligned the text of both section and make one broad section named “Data collection tools and variables”. Firstly, write about tools. Then under this broad section, write outcome variables’ and independent variables’ sub section.

**Response**- *Thank you for the comment. We have done accordingly.*

10. Line 206-209: VIF>10 is mostly useful for continuous independent variables. Please revisit other standard way for detecting multi-colinearity for categorical variables.
**Response**- *Thank you for the comment. We used Chi square test for categorical variables, and all variables significant at 5% level of significance in bivariate analysis were considered for multivariate analysis.*

11. Line 219-220: Which indicators or scale was used to calculate this Cronbach’s alpha?

**Response**- *Thank you for the comment. To check internal consistency in the mother’s autonomy scale, Cronbach’s alpha was used.*

Results

1. Line 198-206 and line 258-291: I have some major observations in the data analysis. I think data analysis did not conducted properly as written in Statistical analysis section.

Here there are four main outcome variables and many independent variables. Firstly, chi-square test and bi-variate logistic regression need to do for each outcome variable with all relevant independent variables. Then all significant independent variables found in chi-square test or bi-variate logistic analysis need to insert in multivariate regression. You may consider the multi-collinearly or overlapping nature of independent variables. For all 4 outcomes, you have to repeat this analysis. The final interpretation should be based on adjusted analysis. Please see some format of other published articles. If you don’t want to put the elaborate analysis in the main manuscript then you can only put the result of adjusted analysis in the manuscript. But you have to put the elaborate analysis in a supplementary file. My main concern is, some most important individual or household level determinants (e.g., mother age, education, employment, number of child/parity, wealth quintile, ethnicity, family type, food insecurity status, child age, sex etc) may not included in the bi-variate and multivariate analysis. For this reason, the effect sizes between bvariate and multivariate regression did not changed more. Please revise the full analysis.

**Response**- *Thank you for the comment. We have included bivariate analysis as supplementary file (S6).*

2. Table 7: The crude OR and Adjusted OR for ANC and PNC reversed completely. There might be some problem in somewhere. Please consult with a public health statistical expert.

**Response**- *Thank you for the comment. We have revised the analysis.*


Discussion and conclusion

1. Based on the available result, the discussion and conclusion was well written. But full discussion and a conclusion may need to revise based on revised result.

**Response**- *Thank you for the comment. Discussion and conclusion have been revised.*

2. My concluding remarks and suggestion to the authors is that if most of the maternal health utilization factors would not significant in the revised adjusted data analysis, then to change objective and title as general determinant exploration of IYCF practices.

**Response**- *Thank you for the comment. Factors such as maternal education, maternal health service utilization, maternal knowledge, decision making power are still significantly associated with IYCF practices in revised data analysis.*
