# Supplementary material for: Infant and young child feeding practices and its associated factors among mothers of under two years children in a western hilly region of Nepal
Source: PLoS One. 2021 Dec 16;16(12):e0261301. doi: 10.1371/journal.pone.0261301 (PMC8675745; doi:10.1371/journal.pone.0261301)
Supplement: Attachment — Submitted filename: Response to reviewer 28.06.21.docx [file pone.0261301.s007.docx]

Reviewer #1: Congratulontions on your article!

After the suggested changes it is clear and cohesive. Unfortonately missing data could not be included, what could have added more relevance.

The added references made the difference in the discussion.

Reviewer #2: Thanks for submitting the 1st revision of the manuscript. After careful consideration of the result of bivarite and multivariate analysis, and other supplementary materials, I think some additional revision is required to meet the adequate standard of a scientific manuscript. I suggested that authors should review some systematic review of IYCF practices, especially enablers and barriers of IYCF practices in the relevant context. The reflection of these systematic reviews is required throughout the whole manuscript. Authors need to do possible modification wherever possible otherwise need to mention as limitations. I am mentioning some of my specific observations below,


Title

Throughout the manuscript, especially the Result and Discussion, I do not find any special reflection of maternal factors on all IYCF practices. So, I suggest not to use maternal factors in the title, rather mentioning about general determinants or factors of IYCF in western hilly region of Nepal.

Response: *Thank you for your comments. We have modified the title accordingly.*

Abstract
Authors need to revise Abstract according to overall revision of manuscript.
*Response: We have revised the abstract according to revised manuscript.*
Introduction
1. Line 83-89: Please omit the special importance of maternal factors and make it general. Focus on the rationale of IYCF exploration in the hilly area of Nepal.
Materials and methods

*Response: Thank you for the comment. We have modified the introduction accordingly.*

1. Sampling: Generally in Multistage sampling, PPS was done to select 1st stage cluster (municipality), and in second stage fixed number of sample is taken from each 1st stage cluster. This is self weighting sampling and having some statistical advantage. But authors did the opposite (missed to take a statistical opportunity). No matter what, please write, how many rural and urban municipalities are in the Syangja district?

*Response: Thank you very much for the comments. We have already mentioned in methodology that Syangja district has 5 urban municipalities and 6 rural municipalities.*

2. Line 122-28: The details of maternal autonomy questionnaire need to incorporate in “Independent variables” section.

*Response : Thank you very much for your comments. We incorporated it in independent variables section.*
3. Maternal knowledge on feeding practice: How the low, medium and high knowledge is categorized? A combined score of maternal knowledge on different child feeding practice at different age may not have the causal temporal relationship with all IYCF practice. For example, knowledge of breastfeeding may not plausibly improve the dietary diversity practice. So, I suggest not to create a combined knowledge score, rather use them independently as separate independent variable whereas applicable in relevant analysis table.

*Response: Thank you very much for your comments. We have incorporated your suggestion.*
4. Statistical analysis: Refer S6 (Bivariate analysis final) and also tell that the variable found significant in chi-square test included in bivariate and multivariate logistic regression.

*Response: Thank you for your comments. We have mentioned this in statistical analysis section.*

5. Line 196-97: Drop the sentence “Multi-collinearity was assessed before performing logistic regression by Chi-square test”. Multi-collinearity is not problem here because a few independent variables found significant at first stage.
*Response: Thank you for your comment. We have addressed this now.*
Results

1. Authors need to make some more categories of education for understanding the doge response relationship. Make at least 3 categories of consistent with Nepal DHS or other similar literature of Nepal. Why the informal education comes as a separate category? Primarily, I am suggesting 4 categories, i.e., illiterate, informal+primary, secondary, and higher secondary or above. Authors could explore different ordered categories. Also use same education category for all outcome variables’ table.

*Response: Thank you for your comments. We have addressed the comment. We have also used same categories of education in bivariate and multivariate analysis*.

2. Authors need to use the frequency of ANC as category for understanding the doge response. For example, 0 ANC, 1-3 ANC, 4-6 ANC and 7+ANC. Existing literatures emphasized the relationship of ANC number and IYCF practices more.

*Response: Thank you so much for your comments. We have categorized the variable as per your suggestion and used the same in bivariate and multivariate analysis.*

3. Authors should try to use same independent variables wherever applicable for all the tables of S6 (Bivariate analysis final). In table 2 of S6, Sex of the child must be included.

*Response: Thank you for your comment. It has been addressed.*
4. Birth order of last child is an important determinant of most of IYCF practices. Number of child could be a proxy of birth order. The number of child should be re-categorized as 1, 2, 3 and 4+.

*Response: Thank you for your comment. It has been re-categorized.*

5. The order of Yes/No column of all tables of S6 should be unique.

*Response: Thank you so much for your comment. It has been addressed.*

6. Some important determinants of IYCF were not included in the analysis. For example, preterm birth, perceived size of child, lower birth order, parity, household food security, father’s education, maternal employment, exposure to media, family level health promoter’s visit for IYCF promotion and IYCF specific counseling and knowledge development (i.e., whether mother received counseling on timely initiation of breastfeeding, exclusive breastfeeding, timely initiation of complementary feeding, dietary diversity and age specific meal frequency; and whether mother developed the specific knowledge of specific IYCF practice). Not all of these indicators were collected in the survey, but some of their proxy can be used. Authors should mention the reasons for not collecting these important variables in the limitation section and also need to write about the way out using the proxy variables.

*Response: Thank you for your comments. We have addressed this in limitations*.
7. How “Food enough to eat for” variable used in the multivariate analysis. This variable is only applicable for if “Crop production” is Yes. “Crop production” is No will make pair-wise case deletion in the multivariate analysis. Authors could make a composite indicator named “Crop production and food security”. Indicator option: 1. Not produce crop or food not enough for 12 months, 2. Produced crop and food enough for 12 months

*Response: Thank you so much for your comments. This has been addressed accordingly.*
8. Maternal occupation should be categorized as standard order of maternal employment.

*Response: Thank you so much for your comment. We have classified the occupation accordingly.*
9. The effective sample size of exclusive breastfeeding analysis is low. Authors need to write in limitation section.

*Response: Thank you for your comment. We have mentioned it as limitation.*
10. Line 232-33 in 1st revised version: 91.9% become duplicated.

*Response: Thank you for your comment. This has been corrected.*
11. Line 238-43: Write the applicable age range of child.

*Response: Thank you for your comment. The standard age categories to assess infant feeding practices was used*
12. Table 4, 5, 6 and 7: Add p-value of Crude OR.

*Response: Thank you for your comment. We have added it.*
13. Table 4, 5, 6 and 7: Write uniquely Crude OR or Unadjusted OR, also Explanatory variable or Variable.

*Response: Thank you for your comments. We have now written it accordingly*.
14. For Crude OR, Adjusted OR and 95% CI, use 1 digit decimal rounding; and for p-value, use 3 digit decimal rounding. Adequate rounding will help the readers to follow easily.

*Response: Thank you for the comment. We have corrected it.*
15. Table 6: The crude OR of Growth monitoring and Nutrition counseling need to recheck and corrected. This is not matching with the proportion of bivariate analysis of S6.

*Response: Thank you for your comment. We have addressed it.*
16. Table 7: The crude OR of PNC visit also need to recheck and corrected. This is also not matching with the proportion of bivariate analysis of S6.

*Response: Thank you for your comment. We have addressed it.*
17. Line 248-78: Table 4, 5, 6 and 7 are the subsequent analysis of S6. At the beginning of write-up, authors need to add an introductory paragraph noting the analysis sequence of S6 to Table 4, 5, 6 and 7.

*Response: Thank you for your comment. We have added a line in the results section.*

Discussion
In discussion, the reflection of all IYCF papers conducted in Nepal must be included. I found some more IYCF papers of Nepal than authors referenced in the discussion. The reasons of similarity or dissimilarity of the result should be convincingly interpreted. Next to the Nepalese IYCF literatures, authors should consider the IYCF papers of South Asian countries, and then if required, need to consider the African, Latin American and East asian context. Authors should explore the recent systematic reviews of South Asian countries. Some of my specific comments of discussion are,

1. From my knowledge, there are sufficient contextual differences among the geographical regions of Nepal, and the context of this study is especially focused in western hilly area. If all region of Nepal is similar then there is no need of this separate study. So, in comparison and result interpretation the differential contextual issue always needs to keep in mind. The result interpretation and programmatic recommendation always need to be concentrated in western hilly area of Nepal.

*Response: Thank you for your comment. We have modified it accordingly.*
2. Line 280-85: The sample size of currently breastfeeding and timely initiation of breastfeeding is not less. What is the programmatic implication of such high rate of breastfeeding in the hilly area of Nepal or Nepal in general? Is this normal secular trend? Is the current government and non-government policy and program is doing well? Is there any future programmatic recommendation? Review more relevant literatures and add logical insight.

*Response: Thank you for your comment. We have modified it accordingly*

3. Authors also need to discuss about the proportion of Initiation of complementary feeding, Minimum dietary diversity and Minimum acceptable diet. Authors also need to generate some programmatic recommendations.

*Response: Thank you for your comment. We have modified it accordingly*

4. Line 286-94: Compare the results with Nepal’s literatures first, then the country of South Asia, if unavailable then can be compared with other countries. What is the reason that nuclear family more likely to initiate breastfeeding on time? Did the Colombian study mention any reason?

*Response: Thank you for your comment. We have modified it accordingly*
5. Line 295-98: Lower than the national average may be only due the low sample size. Were the Cameroon and North West Ethiopia’s studies’ EBF prevalence also lower than their National EBF prevalence? If yes then this reference can be used, otherwise need to delete. The language also need to change, so that we can be understood the similarly of situation that the studies encountered similarly.

*Response: Thank you for your comment. We have modified it accordingly*
6. Line 314-325: Reduce the text. Drop the similar sentence. Inverse association need not to mention here. If mentioned then the reason also need to mention. “Mothers with higher autonomy received more advice about optimal breastfeeding while attending antenatal care.”—Is this really true? How is it? Add reference. Otherwise delete.

*Response: Thank you for your comment. We have modified it accordingly*
7. Line 326-33: Wealth quintile is most important factor found of timely initiation of complementary feeding in most of the similar literature. Why wealth quintile is not a factor for this target population? Authors need to try to explain.

*Response: Thank you for your comment. We have modified it accordingly*
8. Line 332-33: “Considering the sociocultural practices of Nepalese society, female children are introduced early with complementary feeding.”---Need correction

*Response: Thank you for your comment. We have corrected it.*
9. Line 334-51: The non-association of wealth quintile needs to explain. The negative association of maternal autonomy of Benin and Nigeria need not to mention.

*Response: Thank you for your comment. We have modified it accordingly*
10. Line 299-351: In addition to the comparing result and interpretation, some practical and feasible way forward recommendations for nutrition policy, programming and intervention for Nepal or hilly area of Nepal are required. Authors need to include some specific way forward recommendations.
*Response: Thank you for your comment. We have modified it accordingly*

Conclusion

1. The summary of specific recommendations of discussion need to include in Conclusion section.
*Response: Thank you for your comment. We have modified it accordingly*
2. Finally, after the revision, I suggest a 3rd person’s language edit to improve the standard of language.

*Response: Thank you for your comment. Language editing has been performed.*
