# Supplementary material for: Infant and young child feeding practices and its associated factors among mothers of under two years children in a western hilly region of Nepal
Source: PLoS One. 2021 Dec 16;16(12):e0261301. doi: 10.1371/journal.pone.0261301 (PMC8675745; doi:10.1371/journal.pone.0261301)
Supplement: Attachment — Submitted filename: Response to Reviewers 06.11.21.docx [file pone.0261301.s008.docx]

Introduction

1. Line 55-56: “Appropriate infant and young child feeding practices help to prevent almost of all under-five deaths.” --- Please add citation with appropriate reference.

Response – Thank you so much for your comment. We added the citation.

2. Line 65-66: “The government of Nepal has developed and implemented different acts, policies, strategies, and programs to improve infant feeding practices.” --- Please add citation with appropriate reference.

Response – Thank you so much for your comment. We added the citation.

3. Line 75-67: “Maternal factors such as decision-making capacity, education, knowledge, and maternal health services utilization are important factors for child feeding practices.” --- Please add citation with appropriate reference.

Response – Thank you so much for your comment. We added the citation.

4. Line 81-83: Write in simple language mentioning the prevalence of plain and hilly area. Your sentence structure sounds that prevalence of hilly area is lower than plain area!

Response – Thank you so much for your comments. We have written it in simple language.
Materials and methods

1. Line 96-101: Difficult to follow. Write hierarchical and simple language.

Response – Thank you so much for your comments. We have now made it clear.

2. Line 171-174: Add score of reliability of scale here not in Data Quality control section.
Results
Response – Thank you so much for your comments. We added reliability score.

1. Line 214: “26.20 ± 4.148”--- Make 1 decimal point. Do it throughout manuscript except p-value.

Response – Thank you so much for your comments. We have corrected it.

2. Line 229-30: “Less than half (47.6%) of children were exclusively breastfed.” --- Mention the age range of denominator of this proportion for better understanding.

Response – Thank you so much for your comments. We have added the age range.

3. Line 230-31: “More than half (53.3%) of children were initiated with complementary feeding timely. Similarly, nearly half (49.4%) of children were fed according to recommended.” --- Mention the age range of denominator of this proportion for better understanding.

Response – Thank you so much for your comments. We added the age range.

4. “Instutional” spelling should be corrected in Table 6 and Table 7.
Response – Thank you so much for your comments. We have corrected it.

Discussion

1. Line 284-86: “This would suggest that more efforts are needed on improving the quality of maternal and neonatal health care services at health institutions.”--- What kind of effort can be given to initiate breastfeeding within one hour after delivery?

Response – Thank you so much for your comments. We have added the points in discussion.

2. Line 288-88: “Mothers who were from nuclear family were more likely to initiate breastfeeding on time.”--- Please explain why

Response – Thank you so much for your comments. We have added the possible reason.

3. Line 294-96: “This variation might be due to the socio-economic status of the participants, access to a health facility, and sample size compared to this study.”--- This effective sample size of EBF is 103. This small sample size is incapable to capture variation socio-economic status and access to a health facility. So, emphasize the small sample size as only limiting factor and discard socio-conomic status and access to the health facility.

Response – Thank you so much for your comments. We have corrected it.

4. Line 298-302: By definition of EBF is considered 24 hours before interview. How these facility level speculations are linked with EBF within last 24 hours?

Response – Thank you so much for your comments. We have corrected it.

5. Line 296-308: The factor determination of EBF is seriously flawed by the small sample size. So, extended discussion and stronger recommendation is not applicable. For EBF, I only prefer mentioning the result and telling the consistency of literature in similar settings. IF inconsistent findings found then tell about the small sample size as a probable reason.

Response – Thank you so much for your comments. We have corrected it.

6. Line 343-45: Recommendation of further research need to add with the higher sample size (especially for the narrower age range) with other applicable robust designs.

Response – Thank you so much for your comments. We have corrected it.

7. Line 346-47: “In this study, only six indicators were used to assess the feeding practices of children.”--- Is there any more WHO indicators to assess the IYCF? Mention about the independent variables that you missed in the questionnaire.

Response – Thank you so much for your comments. We have corrected it.
Conclusion

1. Line 362-64: “We also need to address the service quality and utilization of maternal health services which is considered to be an important determinant of child feeding practices.”--- In this study service quality is not measured.

Response – Thank you so much for your comments. We have removed the sentence on service quality.
